# Incomplete Freund’s adjuvant reduces arginase and enhances Th1 dominance, TLR signaling and CD40 ligand expression in the vaccine site microenvironment

**DOI:** 10.1136/jitc-2020-000544

**Published:** 2020-04-28

**Authors:** Karlyn E Pollack, Max O Meneveau, Marit M Melssen, Kevin T Lynch, Alexander F Koeppel, Samuel J Young, Stephen Turner, Pankaj Kumar, Katia Sol-Church, Ileana S Mauldin, Craig L Slingluff Jr

**Affiliations:** 1 Surgery, University of Virginia, Charlottesville, Virginia, USA; 2 Biology, University of Virginia, Charlottesville, Virginia, USA; 3 Office of Research Cores Administration (ORCA), University of Virginia School of Medicine, Charlottesville, Virginia, USA; 4 Public Health Sciences, University of Virginia School of Medicine, Charlottesville, Virginia, USA; 5 Biochemistry and Molecular Genetics, University of Virginia, Charlottesville, Virginia, USA; 6 Pathology, University of Virginia School of Medicine, Charlottesville, Virginia, USA

**Keywords:** adjuvants, pharmaceutic, immunogenicity, vaccine, melanoma, arginase, cytokines

## Abstract

**Background:**

Immunogenicity of cancer vaccines is impacted by adjuvants and schedule, but systematic assessments of their effects have not been performed. Montanide ISA-51, an incomplete Freund’s adjuvant (IFA), is used in many vaccine trials, but concerns have been raised about negative effects in murine studies. We found in humans that IFA enhances systemic immune responses and that repeat vaccination at one site (same site vaccination (SSV)) creates tertiary lymphoid structures (TLS) in the vaccine site microenvironment (VSME). We hypothesized that vaccination with peptides+IFA+pICLC or SSV×3 with peptides in IFA would create an immunogenic milieu locally at the VSME, with activated dendritic cells (DC), TLS-associated chemokines and a Th1-dominant VSME.

**Methods:**

Biopsies of the VSME were obtained from participants on two clinical trials who were immunized with multiple melanoma peptides (MELITAC 12.1) in adjuvants comprising IFA and/or the TLR3-agonist pICLC. Biopsies were obtained either a week after one vaccine or a week after SSV×3. Controls included normal skin and skin injected with IFA without peptides. Gene expression analysis was performed by RNAseq.

**Results:**

VSME samples were evaluated from 27 patients. One vaccine with peptides in pICLC+IFA enhanced expression of CD80, CD83, CD86 (p<0.01), CD40 and CD40L (p<0.0001) over normal skin; these effects were significantly enhanced for SSV with peptides+IFA. Vaccines containing pICLC increased expression of TBX21 (T-bet) but did not decrease GATA3 over normal skin, whereas SSV with peptides in IFA dramatically enhanced TBX21 and decreased GATA3, with high expression of IFNγ and STAT1. SSV with peptides in IFA also reduced arginase-1 (ARG1) expression and enhanced expression of TLR adapter molecules TICAM-1 (TRIF) and MYD88. Furthermore, SSV with IFA and peptides also enhanced expression of chemokines associated with TLS formation.

**Conclusions:**

These findings suggest that SSV with peptides in IFA enhances CD40L expression by CD4 T cells, supports a Th1 microenvironment, with accumulation of activated and mature DC. Increased expression of TLR adaptor proteins after SSV with peptides in IFA might implicate effects of the skin microbiome. Reduced ARG1 may reflect diminished suppressive myeloid activity in the VSME.

**Trial registration number:**

(NCT00705640, NCT01585350).

## Background

Cancer vaccines can expand immune responses to tumor antigens; however, their activity depends on an effective immunological adjuvant. Newer adjuvants include toll-like receptor (TLR) agonists, saponins, RNA and nanoparticles.^1^ More traditional adjuvants include incomplete Freund’s adjuvant (IFA), administered as a water-in-oil emulsion. A more contemporary formulation of IFA, Montanide ISA-51 (Seppic, Paris, France), has better-characterized components than earlier formulations, with toxicity profiles similar to those of other promising adjuvants.^2^ IFA continues to be used in veterinary vaccines.^3^ Montanide ISA-51 emulsions have been used in humans in clinical trials of new influenza vaccines,^4–8^ and in a lung cancer vaccine licensed in Cuba.^9^ In prior studies, others and we have reported high rates of CD8^+^ and CD4^+^ T-cell responses to peptide vaccines for cancer administered in IFA emulsions.^10–12^ High rates of tumor regression have also been observed after vaccination with an HPV peptide vaccine using IFA as an immunological adjuvant.^13^ Thus, human experience supports the use of IFA as a vaccine adjuvant.

On the other hand, murine studies have raised concerns about IFA, showing better T-cell responses with a TLR agonist plus an agonistic CD40 antibody (Ab).^14^ Considering this finding, we performed a clinical trial in humans, testing each of two TLR agonists, with or without IFA. An agonistic CD40 Ab was not available for use; so, as an alternative, the vaccine included an antigen that activates CD4^+^ T cells^10 11^ to induce CD40L and thus to activate CD40 on DC. In contrast to what might have been expected with the murine data, we found that adding IFA significantly *enhanced* both the magnitude and persistence of antigen-specific CD8 T-cell responses.^15^ Differences in the impact of IFA in the murine model versus in humans may be explained by differences in IFA dose, differences in T-cell reactivity or to species differences. IFA does support T-cell and antibody responses to peptide antigens in humans and remains a viable adjuvant for cancer vaccines.

However, remarkably little is known about the effects of IFA locally in the vaccine site microenvironment (VSME). In prior studies, we evaluated the VSME for immune cell infiltrates by immunohistochemistry and found dense aggregates of T cells many of which are retained >6 weeks after the last vaccine.^16 17^ We also found by flow cytometry that T cells infiltrating the VSME induced with IFA and peptide represent higher proportions of total T cells than in the blood. On the other hand, we found that these cells were less functional in an ELIspot assay than circulating vaccine-induced T cells and that they overexpressed integrins that may mediate their retention in peripheral tissues.^18^ These findings, and others, raised concern that the VSME induced with IFA may not support a favorable T-cell response. However, the outcome of our Mel58 clinical trial showed that adding IFA to TLR agonists enhanced the frequency, magnitude, and persistence of T-cell responses. Thus, a goal of the present study was to assess whether expression of immune-related genes in the VSME of patients on that trial (Mel58) and one prior trial (Mel48) may explain the favorable immunogenicity associated with IFA in humans.

In addition to the selection of adjuvant, the timing and route of vaccination may impact immunogenicity. Prior work has shown that immunogenicity of peptide vaccines can be enhanced by increased frequency of vaccine administration.^12^ We have found that vaccines administered in the skin (half-intradermal, half-subcutaneous) induce T-cell responses in 80%–100% of patients, and that particularly strong T-cell responses were observed when vaccines were administered at the same site each week (same site vaccination (SSV)).^10 11 19^ Interestingly, SSV with peptides and IFA can induce development of tertiary lymphoid structures (TLS) in the VSME.^16^ TLS in other tissue sites support ongoing immune responses to local antigens, and they contain mature dendritic cells (DC), aggregates of T and B cells and specialized vasculature resembling high endothelial venules, all of which have been induced in the VSME with SSV×3. DC activation and maturation are crucial for optimal antigen presentation, yet vaccine adjuvants have not been compared systematically for their ability to enhance DC activation in the VSME. Classically, these processes are supported by CD40 ligation, which is provided naturally by CD40L on activated CD4 T cells.^20 21^ However, it is not known whether vaccines induce accumulation of CD4^+^ CD40L^+^ T cells in the VSME where they can interact directly with DC to support their maturation. We hypothesized that CD40L expression and DC activation would be enhanced in the VSME after repeated immunization with peptides in IFA, as well as by addition of TLR agonists. Another goal of the present application was to assess whether the same conditions that induce TLS also would induce expression of chemokines implicated in TLS formation.^22 23^

In prior immunohistochemistry (IHC) studies of the VSME, we found early dominance of GATA3^+^ cells over Tbet^+^ cells after one vaccine with IFA, suggesting a Th2-dominant VSME,^18^ which arguably is not ideal for supporting T-cell responses. However, SSV increased Tbet^+^ cells (Th1).^18^ Those studies used only single-color IHC and, thus, were limited in scope. In the present study, we hypothesized that SSV×3 with IFA would enhance expression of genes associated with a Th1-dominant microenvironment. We also performed exploratory analyses of other changes in the VSME, and report evidence for TLR signaling and downregulation of arginase I expression with IFA SSV×3. This work is enabled by prior collection of VSME biopsies from patients on the Mel48 and Mel58 clinical trials, which collected tissue from the VSME at multiple time points. These findings should contribute to a better understanding of the effects of IFA and TLR agonists in the VSME.

## Methods

### Patients and trials

Samples for this study were obtained from patients enrolled in the Mel48 and Mel58 clinical trials at the University of Virginia, for which primary immunological findings have been reported.^15 17 18^ For both trials, each peptide vaccine MELITAC 12.1 consisted of 100 μg of each of 12 melanoma peptides recognized by CD8 T cells (12MP)^19^ plus 200 μg of a tetanus toxoid helper peptide (Tet Tet).^24^ Briefly, for Mel48, patients with resected stage IIB–IV melanoma were randomly assigned to two study groups, each with five subgroups. All participants were vaccinated with MELITAC 12.1 emulsified in IFA (Montanide ISA-51, Seppic) in one extremity, half-subcutaneously and half-intradermally, on days 1, 8, 15, 29, 36 and 43, along with a second immunization at a different skin site, where that immunization was either adjuvant only (group 1) or MELITAC 12.1+adjuvant (group 2). For the present study, we have vaccine site biopsies taken at week 0 (prevaccine: groups 1A, 2A), week 1 (groups 1B, 2B) or week 3 (groups 1C, 2C). In the Mel58 trial, eligible patients were vaccinated with MELITAC 12.1, mixed with either of two TLR agonists (polyICLC (Hiltonol, Oncovir, Washington, District of Columbia, USA), or endotoxin (lipopolysaccharide (SAIC-Frederick, Frederick, Maryland, USA))), with or without IFA.^15^ Each immunization was administered on one skin location, and subsequent immunizations were given in different skin sites (rotating vaccine sites) on days 1, 8, 15, 36, 57 and 78. One week after the first vaccine, patients underwent biopsy of the vaccine site. For the present report, vaccine-site biopsies from patients immunized with the combination of MELITAC 12.1 and polyICLC (1 mg), with and without IFA, were analyzed. All patients were studied following informed consent, and with Institutional Board Review (IRB) (HSR-IRB 13498 and 15781, respectively) and Food and Drug Administration approval.

### RNA extraction and library preparation

Total RNA was isolated from cells collected at the vaccination sites detailed above. RNA extraction was performed using the RNeasy Lipid Tissue MiniKit (Qiagen), according to manufacturer’s recommendations. RNA samples were processed for library preparation using the NEBNext Ultra II Directional RNA Library Prep Kit (Illumina, San Diego, California, USA), according to validated standard operating procedures established by the UVA School of Medicine’s Genome Analysis and Technology Core. Briefly, total RNA was used to isolate mRNA, using NEBNext Poly(A) mRNA Magnetic Isolation Module (New England Biolabs, Ipswich, Massachusetts, USA), followed by fragmentation and first-strand and second-strand cDNA synthesis and fragmentation, as recommended by the manufacturer. The resulting cDNA was end-repaired, adenylated and then subjected to sequence adapter ligation. The final purified libraries were quantified and sized using the Invitrogen Qubit 3 Fluorometer (ThermoFisher Scientific, Waltham, Massachusetts, USA) and Agilent Technologies 4200 TapeStation (Agilent Technologies, Santa Clara, California, USA).

### Next-generation sequencing run and QC

RNA sequencing was performed by the UVA genomics core using the Illumina NextSeq 75 bp High Output sequencing kit reagent cartridge in conjunction with the Illumina NextSeq 500 (Illumina; 75 cycle, single read sequencing), according to the standard manufacturer-recommended procedure. Samples were randomized into four groups and run sequentially on the Illumina NextSeq 500 for single-end sequencing. After transfer to the Illumina Base Space interface, the quality of the runs was assessed by the number of reads in millions passing filter and the per cent of indexed reads. All runs passed the rigorous Illumina run quality control with an average of 94% passing filter, 94% Q30, 38 Gb per run, 439 million reads per run and 96% index recovery.

### Multiparameter immunofluorescence histology

To test whether CD40L protein was present on CD4 T cells, multiparameter immunofluorescence histology analysis was performed; 4 µm thick sections were cut from formalin-fixed paraffin-embedded vaccine site tissue specimens, and human lymph node was used as a positive control. Multispectral staining was performed according to the manufacturer’s protocol using the OPAL Multiplex Manual IHC kit, and antigen retrieval buffers (AR) 6 and 9 (Akoya Biosciences, Marlborough, Massachusetts, USA). Staining sequence, antibodies and antigen retrieval buffers were as follows:

AR9, CD40LG (1:50, Sigma Aldrich, St. Louis, Missouri, USA cat#HPA045827) (Sigma Aldrich) Opal620; AR9, CD4 (1:100, clone SP35) (Cell Marque, Rocklin, California, USA) Opal520; AR9, CD8 (1:500, clone C8/144B) (Agilent Technologies) Opal540; AR6, ICOS1 (1:2 k, clone D1K2T) (Cell Signaling) Opal650; AR6, CD20 (1:4 k, clone L26) (Agilent Technologies) Opal570; AR9, CD3 (1:100, clone MRQ-39) (Cell Marque) Opal690 and spectral DAPI (Akoya Biosciences).

Stained slides were mounted using prolong diamond antifade (Life Technologies, Carlsbad, California, USA) and scanned at 10× magnification using the PerkinElmer Vectra 3.0 system and Vectra software (Akoya Biosciences). Regions of interest were selected in Phenochart software, and 20× magnification images were acquired with the Vectra 3.0 system. These images were spectrally unmixed using single stain positive control images in the InForm software (Akoya Biosciences). Images were analyzed using HALO software (Indica Labs, Albuquerque, New Mexico, USA).

### RNA sequencing and statistical analysis

RNAseq reads were assessed for quality using FastQC. Transcript abundances were then quantified against the human reference genome (Gencode v28 Transcripts, Ensembl GRCh38) using Salmon^25^ and read into the R statistical computing environment as gene-level counts using the tximport package.^26^ The DESeq2 Bioconductor package^27 28^ was used to normalize for differences in sequencing depth between samples (using the default median-of-ratios method), estimate dispersion and fit a negative binomial model for each gene. The Benjamini-Hochberg False Discovery Rate procedure was then used to re-estimate the adjusted p values. As an indirect validation of the RNAseq findings, linear regression was evaluated between previously published available IHC data^17^ from the vaccine sites and RNAseq data (online supplementary figure 1). All statistical analyses and data visualization, including GAGE,^29^ KEGG^30^ and gene set enrichment analysis (GSEA)^31^ were done using the R statistical computing environment and GraphPad Prism 8 (GraphPad Software, San Diego, California, USA).

10.1136/jitc-2020-000544.supp1 Supplementary data

## Results

To assess effects of IFA and pICLC with and without peptides, we performed gene expression profiling on biopsies of vaccine sites obtained from 27 patients, including those enrolled in the Mel48 and Mel58 clinical trials (table 1).

**Table 1:** Table 1Vaccine site treatment conditions by clinical trial

Skin biopsy Sample ID	N	Vaccine at VSME biopsy site	# vaccines at VSME	Date of biopsy	Clinical trial	Study arm/group
Control: normal skin	3	None	0	0	Mel48	1A, 2A
IFA, w1	3	IFA only	1	7	Mel48	1B
IFA+P, w1	5	MELITAC 12.1+IFA	1	7	Mel48	2B
pICLC+P, w1	4	MELITAC 12.1+polyICLC	1	7	Mel58	2A (V0)
pICLC+IFA+P, w1	4	MELITAC 12.1+IFA+polyICLC	1	7	Mel58	2C (V6)
IFA, w3	4	IFA only	3	22	Mel48	1C
IFA+P, w3	4	MELITAC 12.1+IFA	3	22	Mel48	2C
Total	27					

Number of evaluated samples with each vaccine site treatment condition.

IFA, incomplete Freund’s adjuvant; p, peptide; pICLC, poly ICLC; w1, week 1; w3, week 3.

### Dendritic cell activation and maturation

**Figure 1:**
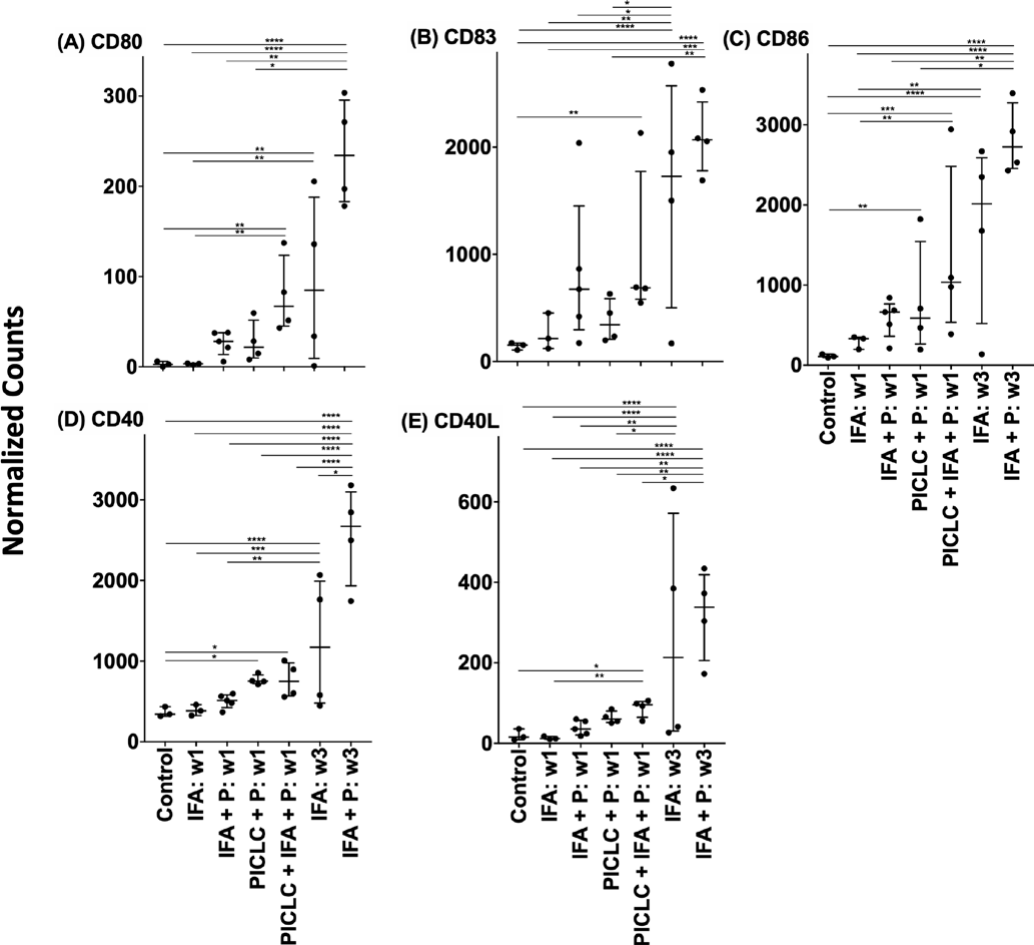
Expression of select genes within the vaccination site microenvironment following treatment with different vaccine compositions. Expression data are provided in terms of normalized counts. bars demonstrate median and IQR. n=27. IFA, incomplete Freund’s adjuvant; P, peptide; w, week. *P<0.05, **p<0.01, ***p<0.001, ****p<0.0001.

Having noted formation of TLS that include mature DC after SSV vaccination with peptides in IFA, we hypothesized that this regimen would enhance expression of markers of DC activation (CD80, CD86) and maturation (CD83) in the VSME. One injection with IFA alone did not produce any significant changes in gene expression compared with normal skin (figure 1A–C). Adding peptides to IFA alone induced expression of CD80, CD83 and CD86, which trended non-significantly higher (figure 1A–C). After one vaccine with peptide+pICLC, expression of CD80 and CD83 were not significantly increased over normal skin, but CD86 expression was higher (p<0.01, figure 1C). Vaccines containing polyICLC+IFA+peptides generated a more robust increase in expression than either agent acting alone (p<0.01 vs control for CD80, CD83, CD86), suggesting that the combination of adjuvants, when administered with peptides, support activation and maturation of DC in the VSME at 1 week (figure 1A–C). Interestingly, there were significant increases in all these genes with SSV×3 with IFA alone; however, these markers of DC activation and maturation were most dramatically upregulated after SSV×3 with MELITAC 12.1 in IFA (figure 1A–C).

### CD40L and CD40 expression following single and repeat vaccination

**Figure 2:**
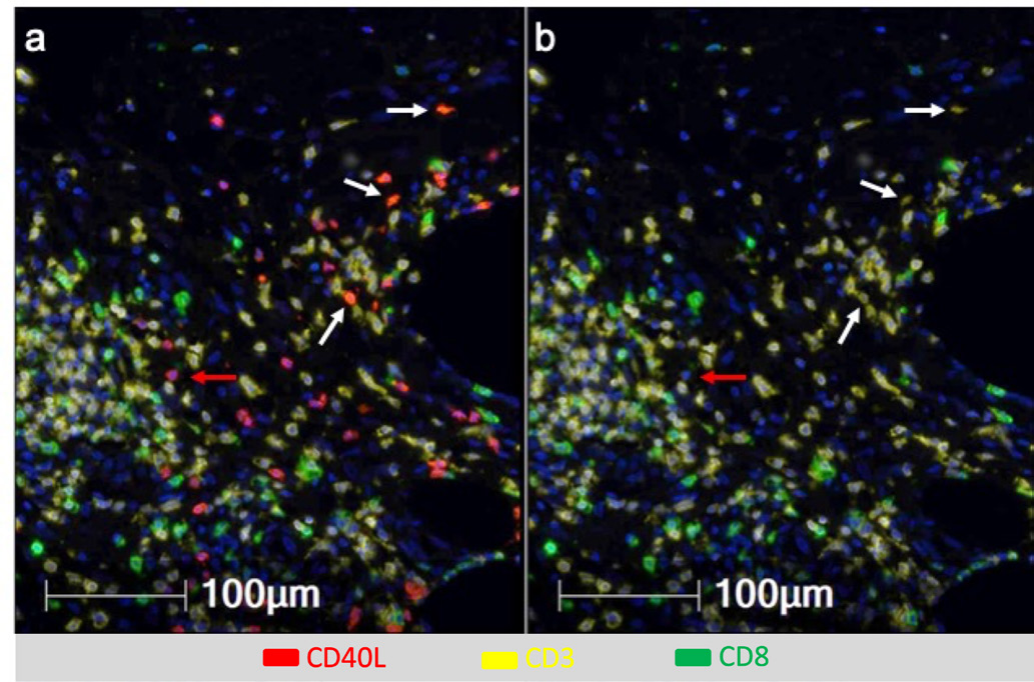
Multiparameter immunofluorescence histology of the vaccine site microenvironment postvaccination with peptides in IFA and pICLC showing co-expression of CD40L and CD3 but not CD8. Selected markers include Dapi (blue), CD40L (red), CD3 (yellow), CD8 (green). (A) Includes CD40L and (B) excludes CD40L. White arrows depict CD3^+^CD8^neg^ T cells co-expressing CD40L and CD3 but not CD8. Red arrows depict cells expressing CD40L but not CD3 or CD8.

DC licensing is mediated through CD40 activation, and can be enhanced by addition of TLR agonists.^32^ Vaccination with a tetanus helper peptide (included in MELITAC 12.1) is effective for stimulating CD4^+^ T-cell responses systemically^11^; thus, we have hypothesized that these vaccines will activate CD4 T cells in the VSME, and that those CD4 T cells would express CD40L. We evaluated CD40 and CD40L expression in the VSME following both single and repeat immunization (figure 1D, E). One vaccine with peptides in pICLC, but not IFA, conferred an increase in CD40 expression, but not of CD40L. Both were increased at 1 week with polyICLC+IFA+peptides. SSV×3 with IFA alone or in combination with peptides produced a dramatic increase in expression of both CD40 and CD40L (p<0.0001 vs control, figure 1D, E). To verify that CD40L protein itself was present on CD4 T cells in the VSME, multiparameter immunofluorescence histology was performed on samples from two patients to assess co-expression of CD40L on CD3^+^ CD8^neg^ cells as an estimate of CD4^+^ cells expressing CD40L protein in the VSME. (CD4 antibody staining was suboptimal; thus, CD3^+^CD8^neg^ staining was used as a surrogate for CD4^+^ cells). A representative sample of the VSME after SSV×3 with pICLC+IFA+peptides is shown in figure 2, which confirms expression of CD40L on multiple CD3^+^ CD8^neg^ T cells.

### Transcription factors and Th1 dominance

Having previously observed a dominance of GATA3 staining over Tbet staining within the VSME following single injection with peptides and IFA,^17^ we conducted a more comprehensive evaluation of the expression of Th1, Th2, Th17 and Treg transcription factors (TBX21, GATA3, RORγt/RORC and FOXP3, respectively), as well as Th1-associated genes for IFNγ (IFNG) and STAT1, following vaccination. We hypothesized that repeat vaccination with peptide in IFA would enhances a Th1 signature compared with one vaccine, resulting in an increase in both STAT1 and IFNG expression.

**Figure 3:**
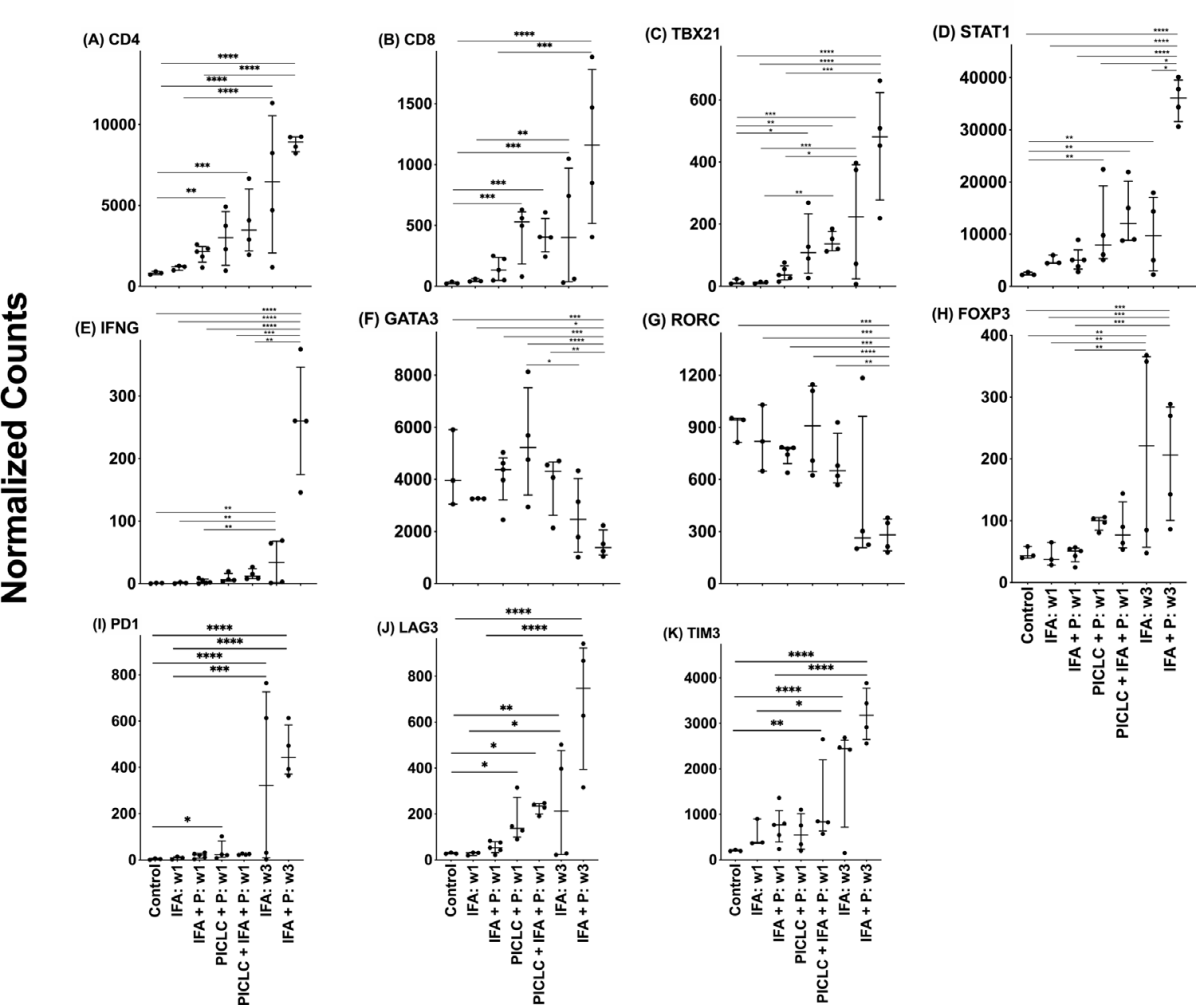
Expression of T-cell markers, transcription factors and markers of exhaustion within the vaccination site microenvironment following treatment with different vaccine compositions. Expression data are provided in terms of normalized counts. Bars demonstrate median and IQR. n=27. IFA, incomplete Freund’s adjuvant; P, peptide; w, week. *P<0.05, **p<0.01, ***p<0.001, ****p<0.0001.

One vaccine with IFA, alone or in combination with peptides, had little effect on expression of these genes. Vaccination with peptides in pICLC, with and without the addition of IFA, resulted in significant recruitment of CD4^+^ and CD8^+^ T cells to the VSME as evidenced by increases in CD4 and CD8 expresssion (figure 3A, B). Peptides in pICLC, with and without IFA, also increased the expression of TBX21 and STAT1, but not IFNG (figure 3C-E) or any of the other transcription factors (figure 3). It was notable that there was baseline expression of GATA3, such that in normal skin, the Th2:Th1 ratios were high (299.34) (figure 3F). However, repeat (SSV×3) vaccination with peptides in IFA induced a dramatic increase in expression of Th1 genes, including TBX21 (p<0.001), STAT1 (p<0.01) and IFNγ (p<0.01), and a significant decrease in GATA3 and RORC compared with most other conditions (figure 3F, G). FOXP3 (p<0.01) expression also increased following repeat vaccination (figure 3H). Interestingly, PD-1 expression remained relatively low in all conditions except in repeat (SSV×3) vaccination with IFA alone and peptide plus IFA (figure 3I). Additionally, LAG3 and TIM3 increased with repeat SSV with peptide plus IFA (figure 3J, K).

### GAGE pathway analysis

**Figure 4:**
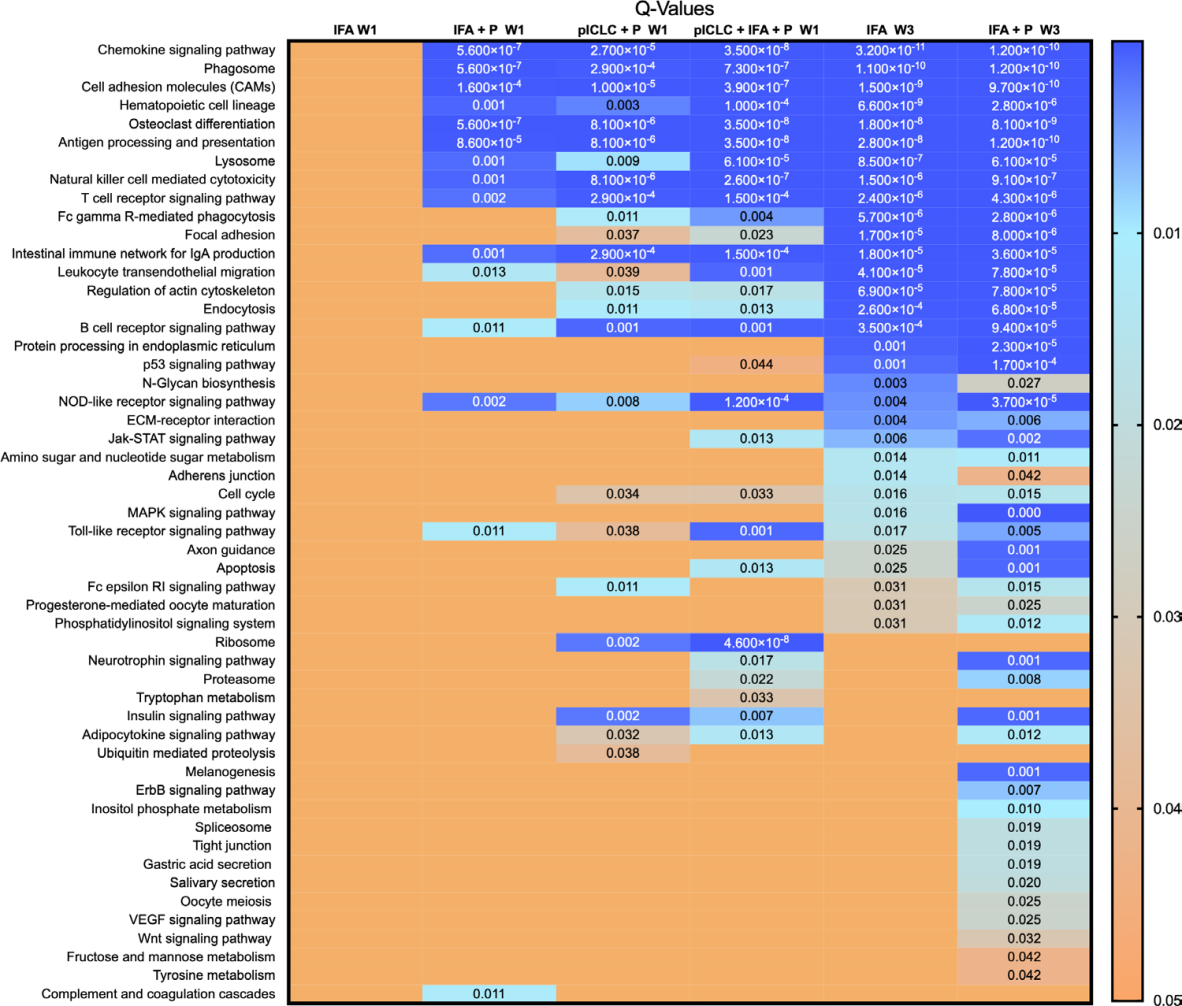
Results of GAGE pathway analysis performed on the complete set of differentially expressed genes under each vaccination condition. Q-values are provided for each enriched pathway under the various vaccination conditions. If a Q-value is not provided, the pathway was not enriched by the associated vaccination condition. IFA, incomplete Freund’s adjuvant; P, peptide; w, week.

To gain better insight into the biological pathways affected by the changes in the VSME, we applied GAGE pathway analysis on the complete set of differentially expressed genes under each vaccination condition. With the exception of single vaccination with IFA alone, all other treatment methods resulted in significant enrichment of 14 identical pathways, which were related to inflammation, chemokine signaling, T-cell and B-cell receptor signaling, antigen processing and presentation and cell migration (figure 4).

Overall, repeat vaccination with IFA affected a higher number of pathways than single vaccination, regardless of composition. SSV with the combination of peptides in IFA conferred the highest impact, leading to significant enrichment of 48 pathways. Most pathways impacted by the other vaccine combinations were included among those enriched by repeat vaccination with peptides in IFA. Exceptions include the ‘Complement and Coagulation Cascade’ pathway, which was significantly enriched only following single vaccination with peptides in IFA, the ‘Ribosome’ pathway, enriched following single vaccination with peptides in pICLC, with and without IFA, the ‘Ubiquitin Mediated Proteolysis’ pathway, affected by single vaccination with peptides in pICLC and the ‘Tryptophan Metabolism’ pathway, enriched only by single vaccination with peptides in pICLC and IFA.

### Arginase-1 expression following vaccination with pICLC and IFA

**Figure 5:**
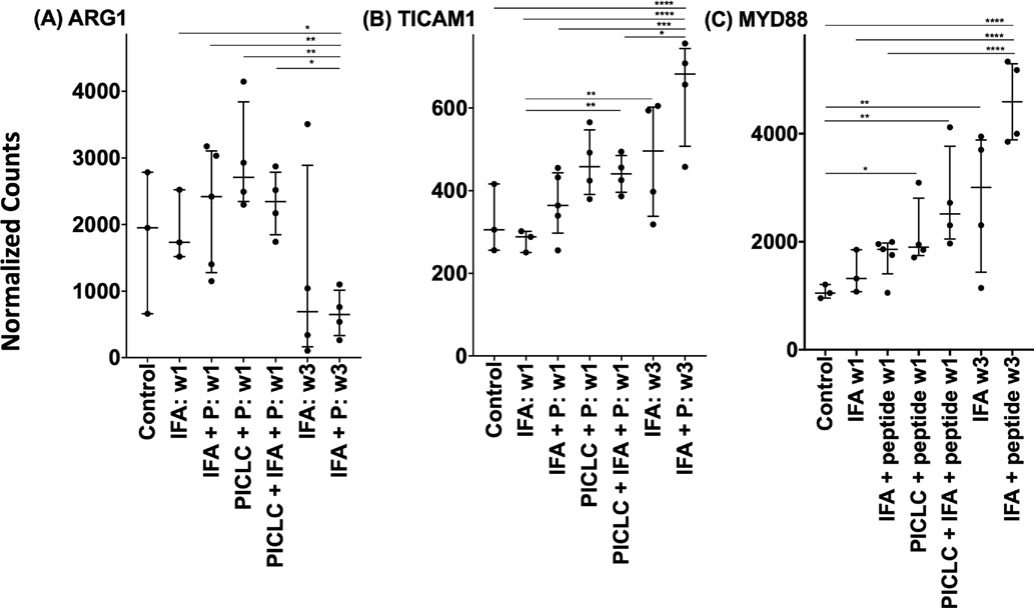
Expression of arginase-1 (A), TICAM1 (TRIF) (B) and MYD88 (C) within the vaccination site microenvironment following treatment with different vaccine compositions. Expression data are provided in terms of normalized counts. Bars demonstrate median and IQR. n=27. IFA, incomplete Freund’s adjuvant; P, peptide; w, week. *P<0.05, **p<0.01, ***p<0.001, ****p<0.0001.

**Figure 6:**
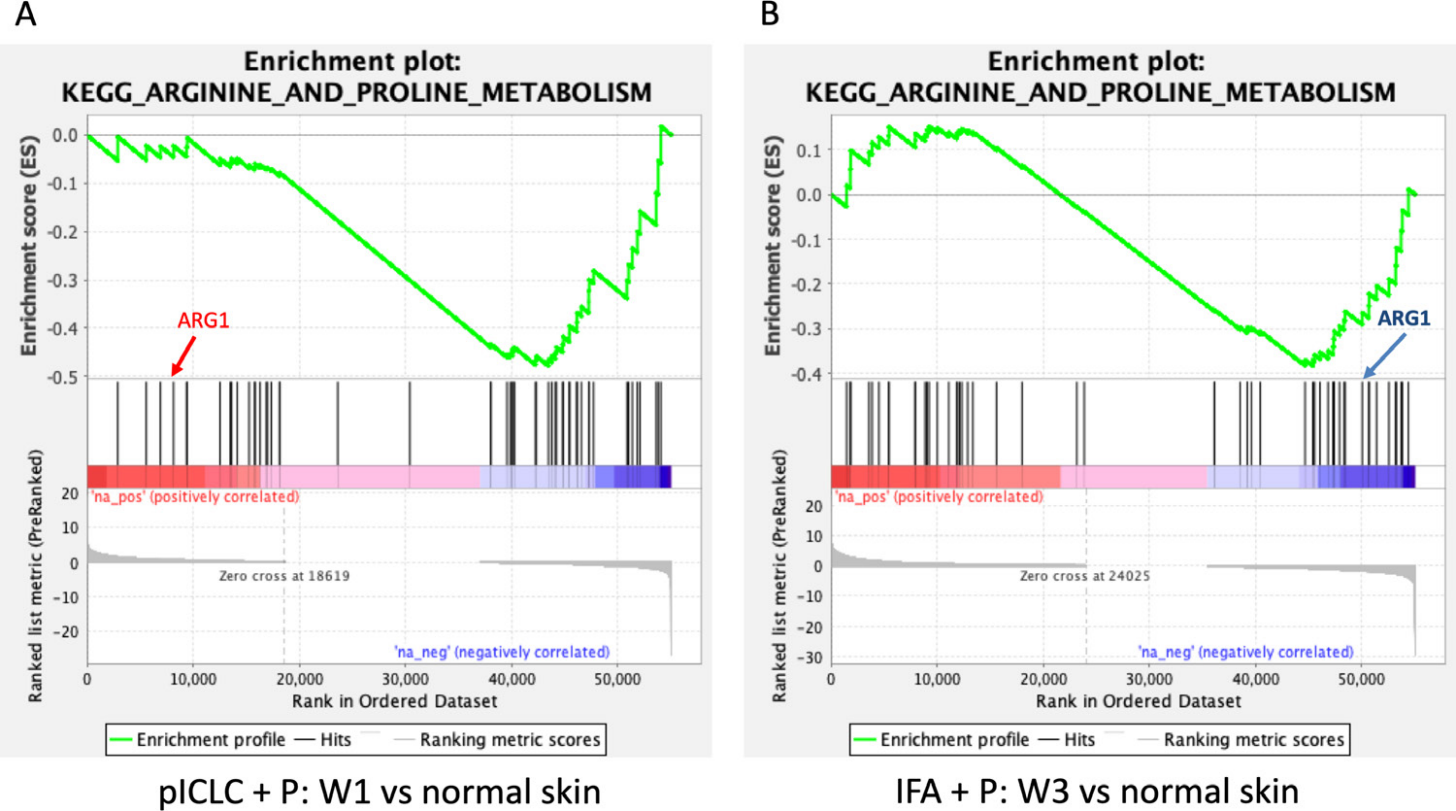
Gene set enrichment analysis highlighting the varied pattern of downregulation of arginine and proline metabolism following single vaccination with peptides in pICLC (A) and repeated vaccination with peptides in IFA (B). ARG1 is not meaningfully downregulated by peptides in pICLC (A) but is strongly downregulated in repeated vaccination with peptides in IFA (B) as shown by its position in the ranked list of genes. Both conditions were compared with normal skin control. Gene set enrichment data were generated using GAGE and included genes from the arginine and proline metabolism pathway. ARG1, arginase-1; IFA, incomplete Freund’s adjuvant; pICLC, poly ICLC; p, peptide.

Myeloid-derived suppressor cells can down-modulate antitumor immune responses,^33^ with the potential to suppress T-cell responses and function, largely through the action of arginase-1 (ARG1). In an effort to characterize better the activity induced by peptide vaccines in IFA with or without polyICLC at the VSME, we analyzed the expression of ARG1 following single and repeat immunization (figure 5A). We found that one vaccine with IFA, either alone or in combination with peptide, has little effect on ARG1 expression. Following three vaccinations with peptide in IFA, expression of ARG1 trends downward and is nearly half that is observed within normal skin (p=0.06, figure 5A). This gene expression is significantly lower than that seen with IFA and peptide at week one (p<0.01) or any other test condition except repeat vaccination with IFA alone. The results of a GSEA depict significant, global downregulation of the arginine and proline metabolism pathway following both single vaccination with peptides in pICLC (figure 6A) and repeat vaccination with peptides in IFA (figure 6B), compared with normal skin. ARG1 itself was not downregulated by peptides in pICLC (figure 6A) but highly downregulated by repeat vaccination with peptides in IFA (figure 6B). Taken together, these results suggest a qualitative difference in ARG1 expression following one vaccine with peptides in pICLC compared with repeated vaccination with peptides in IFA (figure 5A) despite global downregulation of arginine and proline metabolism in both conditions.

### Toll-like receptor pathway signaling

The GAGE pathway analysis revealed that the ‘toll-like receptor signaling’ pathway was significantly perturbed by all vaccine conditions, with the exception of that involving single vaccination with IFA alone (figure 4). Furthermore, within the vaccine conditions that led to pathway enrichment, expression of both MYD88 and TRIF increased, and were within the top 500 most differently expressed genes, when compared with normal skin (data not shown). This was expected since polyICLC has TLR3 agonist activity. However, it is notable that vaccination with peptides+IFA also induced significant changes in TLR signaling. To understand these changes, we have evaluated changes in expression of the key adaptor molecules MYD88 and TRIF (TICAM1). TLR3 signals through TRIF, rather than MYD88; so, we expected TRIF expression to be enhanced when vaccinating with polyICLC. We found that TRIF expression trended higher 1 week after vaccination with polyICLC+peptides, with a significant increase when IFA was also in the vaccines (figure 5B). An unexpected finding was that SSV×3 with peptides in IFA led to dramatic and significant increases in TRIF compared with most other conditions, despite there being no TLR agonist in those vaccine formulations.

There was also a significant rise in MYD88 expression following single vaccination with peptide in pICLC, compared with normal skin, and an even more significant enhancement in MYD88 was observed with peptide+polyICLC+IFA at 1 week (figure 5C). Interestingly, SSV×3 with IFA alone also enhanced MYD88 expression. The most significant increases in MYD88 were observed with SSV with peptides in IFA (p<0.0001).

### Expression of a 12-chemokine gene signature associated with the formation of tertiary lymphoid structures

In prior work, we identified TLS formation within the VSME after repeated vaccination with peptides in IFA.^16^ A 12-chemokine gene signature has been identified as being associated with TLS formation in melanoma^34^; so, we have evaluated this signature to understand possible contributing factors in TLS formation in the VSME. There was little effect on expression of these chemokine genes with one injection of IFA alone (online supplementary figure 2), either alone or in combination with peptide; however, one injection with peptides in pICLC led a significant increase in expression of 7/12 chemokine genes (CCL3, p<0.05; CCL4, CXCL10, CXCL11, p<0.01; CCL5, CCL21, CXCL9, p<0.001). Vaccination with peptides, pICLC and IFA appeared to be the most effective combination after single injection, producing an increase in expression in 10/12 genes (CCL2, CCL3, CCL4, CCL5, CCL8, CXCL9, CXCL10, CXCL11, CCL18, CCL19). Notably, however, this combination did not increase expression of CXCL13, which has been strongly identified as playing a crucial role in TLS formation. In contrast, repeat vaccination with IFA, both alone and together with peptides, generated a dramatic increase in CXCL13 expression, plus an additional 9 (CCL2, CCL3, CCL4, CCL5, CCL8, CXCL9, CXCL10, CCL18, CCL19) or 10 (CCL2, CCL3, CCL4, CCL5, CCL8, CXCL9, CXCL10, CXCL11, CCL18, CCL19) genes, respectively.

10.1136/jitc-2020-000544.supp2 Supplementary data

## Discussion

A goal of vaccine therapy in cancer treatment is to produce strong, durable and antigen-specific T-cell responses. This requires effective vaccine adjuvants, the most optimal of which has yet to be determined. Current research has focused heavily on the use of TLR-agonist-based adjuvants, and the immunogenicity of these agents has been well-demonstrated in both mice and humans.^35–39^ The present study identifies effects of IFA, polyICLC and peptides in the VSME after one or three repeated vaccines, with evidence that SSV with IFA+peptide reduces the ARG1 expression, enhances CD40L expression and DC activation, supports a Th1-dominant VSME and induces a chemokine signature associated with formation of TLS.

IFA is a more traditional water-in-oil emulsion-based adjuvant. Its efficacy within melanoma vaccines has been called into question in recent murine studies, which reported that the adjuvant fosters the recruitment, exhaustion and eventual death of T cells within the vaccination site.^40^ Nevertheless, as we have reported,^15^ the applicability of those findings to humans may be limited by vaccine dosage volume and the extent of a native T-cell response. Methods in the prior murine study^14^ differed from these human trials by adoptive transfer of a large number of antigen-reactive T cells, as opposed to attempting to expand endogenous T cells, and by a much higher dose of IFA delivered per gram body weight (approximately 4 vs 0.01 µL). These and other factors may explain our different findings, which we have discussed in the primary report of the Mel58 clinical trial.^15^ Furthermore, recent human vaccine studies using IFA, conducted both within our laboratory and by others, have been promising, and have demonstrated the ability of IFA to generate a robust and antigen-specific CD8 T-cell response.^10–12^ In light of these data, we sought to understand possible mechanisms behind IFA’s immunogenicity. Here, we focused on the cellular and molecular effects of IFA within the VSME. Our results highlight the ability of IFA to activate DCs and CD4 T cells within the VSME, support a Th1-dominant environment, and potentially reduce myeloid suppression within the vaccination site. We also present evidence suggesting that repeated SSV with IFA-containing vaccines might be more effective than single vaccination, including inducing the formation of TLS within the VSME.

Evidence of TLS within the VSME was previously demonstrated in a subset of participants on the Mel48 trial, by IHC.^17^ Our current data reinforce this notion, as repeated SSV with peptides in IFA significantly increased expression of 11 out of 12 chemokines whose overexpression has been associated with the TLS in melanoma. On the other hand, CCL21 has been defined as an important driver of TLS neogenesis,^23 41^ but its expression was not consistently enhanced with IFA and peptides at week 3.

In support of our previous IHC staining for cellular transcription factors, we found evidence in favor of repeated SSV with IFA generating a Th1-dominant microenvironment. After three vaccinations with IFA alone, expression of TBX21, STAT1 and IFNG all increased significantly, while expression of GATA3 and RORC trended downward. The addition of peptides enhanced these effects, causing a significant decrease in GATA3 and RORC expression compared with normal skin. This finding suggests that the VSME created by SSV with peptides in IFA may support activation and expansion of Th1 cells and cytotoxic T cells. Interestingly, we also found that repeated SSV with peptides in IFA also upregulated PD-1, LAG3 and TIM3. Increased PD-1 may be explained by increased T-cell numbers and/or an increase of PD-1 expression by activated T cells in the VSME. However, taken together, the increase in these three genes may represent exhaustion on at least a subset of T cells in the VSME. We have previously reported that T cells isolated from the VSME after SSV with peptides in IFA were less likely to produce IFNγ in response to their cognate antigen than circulating T cells,^18^ which also suggests that some antigen-reactive T cells in the VSME are exhausted. However, questions remain about whether PD-1, LAG3 and TIM3 are co-expressed on the same T cells and whether exhausted cells represent a major or minor fraction of the antigen-reactive T cells in the VSME. Future single-cell and functional studies are needed in order to answer this question. The enhanced Th1 signatures, including IFNγ, suggest that immune activation remains a dominant feature of the VSME in this setting.

A question still remaining in light of our current work is whether repeated vaccination with peptides, in combination with both IFA and TLR agonists, will be more effective than repeated SSV with only IFA and peptides. As we reported previously, adding IFA to a single vaccine containing peptides in either of two TLR (lipopolysaccharide and pICLC), enhances the CD8 T-cell response rate and persistence compared with vaccines containing TLR agonist alone.^15^ It is possible that combining the two adjuvants in repeated vaccines will produce an even greater immune response. An ongoing clinical trial, Mel63 (NCT02425306), will have samples to evaluate this question in patients receiving repeated SSV with TLR agonist, IFA and peptide.

Another interesting, yet unexpected, finding from our data was the fact that repeated SSV with IFA alone and in combination with peptides, induced marked increases in expression of both MyD88 and TRIF, despite the absence of an exogenous TLR agonist within the vaccine. There was also a dramatic and pervasive increase in signaling through the TLR pathway under all vaccination conditions. Activation of MyD88 and TRIF by the TLRs 1/2, 2/6, 5, 7, 8, 9 and TLR3, respectively, leads to downstream effects including release of cytokines and chemokines that support immune activation and cellular immunity. The activation of these signaling pathways by IFA, alone or in combination with peptides, sparks questions regarding their source of origin. It is possible that the signaling may be a direct effect of vaccination, itself. However, it is also conceivable that the skin microbiome may be responsible. A recent murine study demonstrated that vaccines involving IFA depend on MyD88 signaling for induction of humoral immunity.^42^ While the exact mechanism behind this process remains unknown, the authors of that study hypothesized a role for microbiota-derived signals. Specifically, tissue damage resulting from vaccination may expose commensal bacteria colonizing the surface of the skin and its appendages to TLRs, thus providing a potential trigger for the initiation of pathway signaling. Subsequent DC activation may then perpetuate this process. Future studies will focus on identifying skin microbiota and determining specific changes in TLR expression within the skin in order to understand these findings.

Notwithstanding the promise of TLR-based adjuvants and other novel adjuvants, the data in the present report support the continued evaluation of IFA with cancer vaccines, both as a single agent and in combination with TLR agonists. Despite its long history of use in cancer vaccines, little has been known about the cellular and molecular effects of IFA in the VSME. This study provides new data to understand the effects of vaccines incorporating IFA, and suggest prominent effects of repeated vaccines given at the same site. The finding of TLR signaling after SSV with peptides in IFA raises new questions about the impact that IFA has on the skin microbiota and the regulation of signaling pathways within the VSME, and supporting favorable effects of this adjuvant in humans.
