# Supplementary material for: Incomplete Freund’s adjuvant reduces arginase and enhances Th1 dominance, TLR signaling and CD40 ligand expression in the vaccine site microenvironment
Source: J Immunother Cancer. 2020 Apr 28;8(1):e000544. doi: 10.1136/jitc-2020-000544 (PMC7213888; doi:10.1136/jitc-2020-000544)
Supplement: Supplementary data [file jitc-2020-000544supp001.pdf]

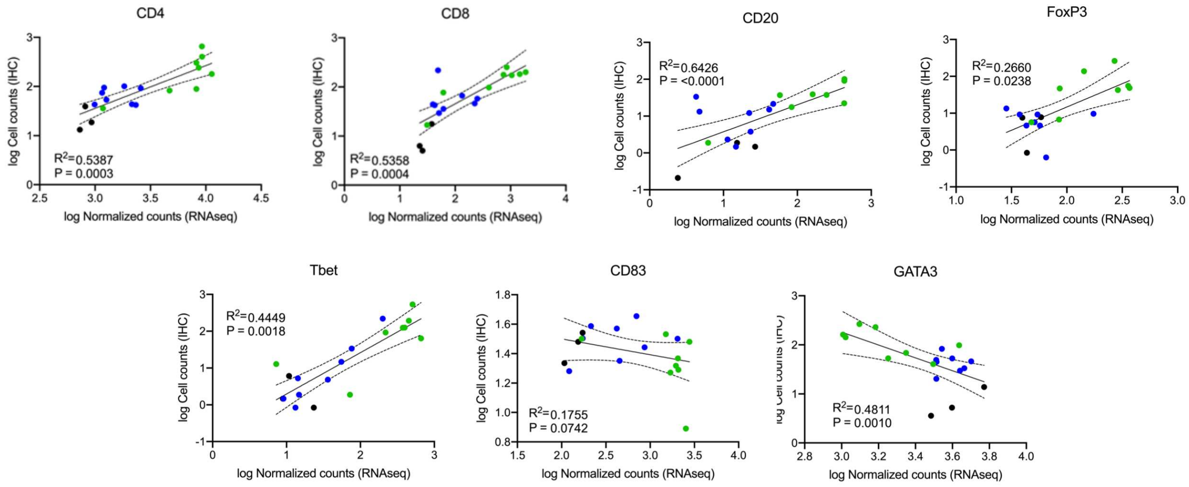

Supplemental figure 1. Linear regression plots of RNAseq log normalized counts vs IHC log cell count data from the Mel48 clinical trial VSME samples. Colors depict clinical trial arm (black: Mel48 1A/2A; blue: Mel48 1B/2B; green: Mel48 1C/2C.)
