# Supplementary figures and images for: Incomplete Freund’s adjuvant reduces arginase and enhances Th1 dominance, TLR signaling and CD40 ligand expression in the vaccine site microenvironment
Source: J Immunother Cancer. 2020 Apr 28;8(1):e000544. doi: 10.1136/jitc-2020-000544 (PMC7213888; doi:10.1136/jitc-2020-000544)

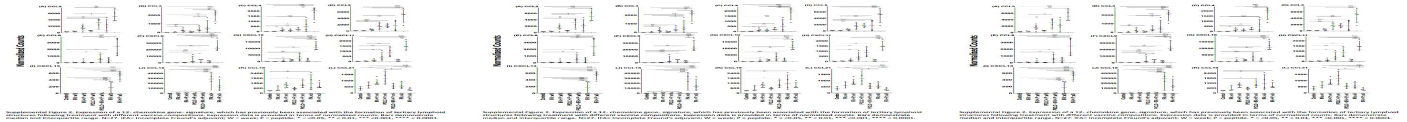

Supplement: Supplementary data [file jitc-2020-000544supp002.pdf]
